# Implant migration and bone mineral density measured simultaneously by low-dose CT scans: a 2-year study on 17 acetabular revisions with impaction bone grafting

**DOI:** 10.1080/17453674.2020.1769295

**Published:** 2020-05-26

**Authors:** Hampus Stigbrand, Keenan Brown, Henrik Olivecrona, Gösta Ullmark

**Affiliations:** a Department of Orthopedics Gävle Hospital, Center for Research & Development, Uppsala University/County Council of Gävleborg, Sweden;; b Department of Surgical Sciences/Orthopedics, Uppsala University, Sweden;; c Mindways Software Inc., Austin, TX, USA;; d Department of Molecular Medicine and Surgery, Karolinska Institute, Stockholm, Sweden

## Abstract

Background and purpose — Early postoperative implant migration predicts failure of joint replacements. Bone mineral density reflects bone quality and bone-graft incorporation. Implant migration and bone densitometry analysis usually require special equipment. We investigated cup migration and bone mineral density changes simultaneously with low-dose CT scans after acetabular revision hip arthroplasty using impaction bone grafting.

Patients and methods — We performed a low-dose CT postoperatively, after 6 weeks, and after 2 years in 17 patients, all revised using impaction bone grafting and a graft-compressing titanium shell in the acetabulum. 6 patients had combined segmental and cavitary acetabular defects. Cup migration was analyzed using CT-based micromotion analysis (CTMA). Bone mineral density was determined in the graft and in surrounding native bone using volumetric quantitative computed tomography (QCT). The bone graft volume was calculated from 3D reconstructions.

Results — At 2 years, the translations were 1.5 (95% CI 0.4–2.6) mm in proximal direction, -0.6 (CI –1.6 to 0.4) in the medial direction and 0.3 (CI 0.0–0.6) in the anterior direction. The mean volume of impacted bone graft was 40 cm³ (CI 28–52). In the graft bone mineral density increased 14% after 6 weeks and 23% after 2 years. There was 1 mechanical failure.

Interpretation — Proximal migration of the acetabular component was low and comparable to previous reports. There was a rapid increase of bone mineral density in the bone graft. Low-dose CT scans make migration analysis and bone densitometry measurements possible in the same setting, offering great diagnostic potential for hip arthroplasty patients.

There is a strong relationship between early prosthetic migration and long-term prosthetic survival for both knee and hip replacements (Pijls et al. 2012). Radiostereometric analysis is the traditional method for exact measurement of prosthetic micromotion. Computed tomography is an alternative that offers comparable precision when using a new software called CT-based micromotion analysis (CTMA, SECTRA AB, Linköping, Sweden). Migration analysis is much easier to perform without specialized equipment. This software defines the surface of the pelvic cortical bone in 2 CT scans taken on 2 different occasions. The software will overlap and match these digital 3D reconstructions in a precise manner (pelvic rigid body). The implant is then defined in the same way in the same examinations. By defining the pelvic bone as the reference, the migration of the implant between the 2 examinations is calculated along the x-, y-, and z-axis. By using the pelvic cortical surface as reference, tantalum markers are no longer a prerequisite for precise definition of the pelvic bone reference (Brodén et al. 2020). This CT-based motion analysis had a precision of 0.07–0.16 mm for translations and 0.10°–0.32° for rotations of acetabular components in a recent study of 24 double examinations with different patient cohorts from 3 Swedish hospitals. 10 patients, with double examinations, from this study were also included in the precision study by Brodén et al. (2020). Prosthetic implants alter the load distribution in peri-prosthetic bone and cause osteopenia—a phenomenon known as stress-shielding (Wright et al. 2001, Bodén et al. 2006) Bone mineral density (BMD) measures only mass of mineral per volume bone tissue. A low BMD is associated with fragility fractures and BMD increases when morselized bone graft is incorporated (Gerhardt et al. 2018). However, the mechanical strength of bone is also dependent on mineralization degree, trabecular architecture, hydroxyapatite crystal size, and collagen properties, therefore BMD is a proxy measurement for bone quality (Fonseca et al. 2014). Dual energy X-ray absorptiometry (DEXA) is currently the most widely used method for clinical measurement of bone mineral density in orthopedics (DeSapri and Brook 2020). However, DEXA screens both cortices and adjacent tissue, whereas quantitative computed tomography (QCT) can study the bone mineral density in a specific region of interest.

We measured postoperative cup migration and bone mineral density simultaneously with serial low-dose CT scans in 17 patients after revision total hip arthroplasty with impaction bone grafting.

## Patients and methods

### Patients and surgery

17 consecutive patients (9 men) scheduled for revision total hip arthroplasty were recruited at the Orthopedic Department, Gävle Hospital from July 2015 to November 2016. The inclusion criterion was acetabular bone loss. Patients with rheumatoid disease, dementia, or patients treated with drugs affecting bone metabolism were excluded. Mean BMI was 28 (23–37) and mean age 73 years (49–87). All patients were revised through a posterolateral approach. Bone defects were classified intraoperatively. 6 patients had combined segmental and cavitary acetabular bone defects according to the AAOS classification. In 5 patients, an acetabular titanium rim plate (Waldemar Link GmbH & Co, Hamburg, Germany) was inserted to repair a segmental defect. A posterior or posterior-superior supporting rim plate was used when the segmental defect compromised cup stability and/or adequate coverage of the implant in this load-bearing area. Medial or anterior acetabular rim reinforcement was not used in this material. Acetabular impaction bone grafting was performed in all cases. A thin, graft-compressing titanium shell, from the same company (Waldemar Link GmbH & Co, Hamburg, Germany), was inserted on top of the graft to enhance compression. A polyethylene cup, size 46–52 mm, with a prosthetic head of 32 or 36 mm (Lubinus Eccentric X-linked acetabular cup, Waldemar Link GmbH & Co, Hamburg, Germany) was cemented inside the titanium shell, for details see Stigbrand et al. (2018). Weight-bearing was allowed immediately postoperatively.

### Radiographic analysis

Low-dose CT scans were performed postoperatively at 6 weeks and 2 years. The polyethylene cup was marked with 1.0-mm tantalum markers. CT-based migration analysis was performed using the CTMA software (SECTRA AB, Linköping, Sweden). Based on 12 double examinations a precision of 0.11–0.14 mm migration was calculated (assuming zero migration). Double examinations were performed within minutes with the patient standing up in between. The surface of the pelvic bone was defined as the skeletal reference body, without tantalum markers in the bone. Precision was defined as repeatability, i.e., the variation in 2 repeated measurements on the same subject under identical conditions over a short period of time.

To get the precision estimates we followed the procedure commonly used for RSA. With n representing the number of patients, we estimated the precision of the method by extracting from a Student’s t-distribution chart of n–1 degrees of freedom the critical value encompassing 95% of the distribution. We then multiplied this by the standard deviation of our double measurements.

**Figure 1.**
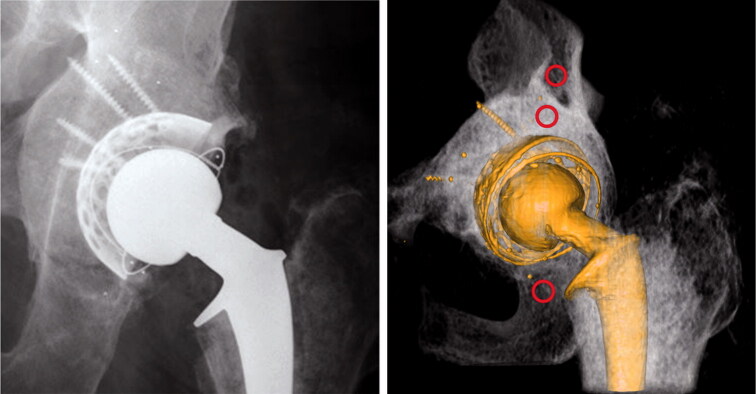
Postoperative images. Note the greater density in the bone graft compared with native bone. The 3 red circles indicate the ROIs of the bone mineral density measurements.

The resulting value is our precision. QCT analysis was performed with Mindways Software (Mindways Software Inc, Austin TX, USA) by defining a volume of 0.4–1.0 cm^3^ in 3 regions of interests: (a) bone graft, (b) native bone cranial to the acetabular bone graft, and (c) native bone in the inferior ramus (Figure 1).

To ensure consistency of sampled volumes over time, the baseline and follow-up studies were analyzed in parallel by the same observer (KB). Distinctive landmarks were viewed on screen simultaneously in each CT study in a set of serial exams, and sample volumes were positioned in each exam with respect to the landmarks.

Care was taken not to include bone cement or screws in the analyzed volumes. A calibration phantom was used to calibrate the CT scan for density measurements. All CT scans were performed on the same Toshiba Aquilion One CT scanner (Canon Medical Systems, Tustin, CA, USA) and radiation dose was approximately 2.3 mSv per examination. For technical reasons, 2 CT scans in 2 cases were excluded. 1 patient had the contralateral hip examined at 2 years and another patient had image disturbances in the QCT analysis.

### Volume measurement

**Figure 2.**
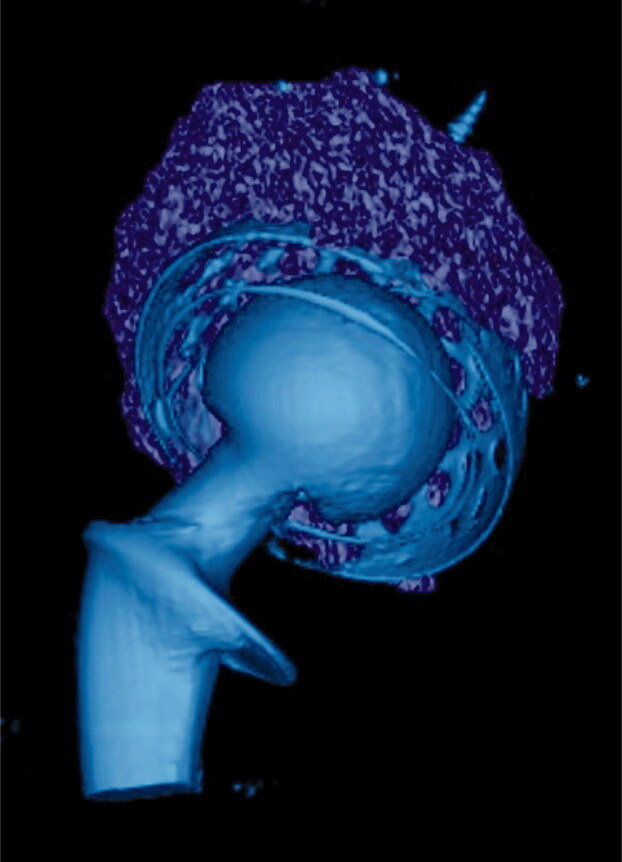
3-D rendering of bone graft volume.

The bone graft volume was measured by defining the bone graft in 3 dimensions. To do this, the border was marked between bone graft and native bone and between bone graft and cement/titanium shell on every 5th CT slide (i.e., every 2.5 mm) (Figure 1). The software Vital Imaging (Miami, FL, USA) approximated the remaining slides. Finally, the grafted volume was controlled and corrected in all 3 dimensions on every slide (Figure 2).

### Clinical follow-up

Clinical examination was performed preoperatively and at 2 years with hip scoring according to Merle D’Aubigne and Postel (Charnley 1979). No patient was lost to follow-up.

### Statistics

17 patients were included. Based on previous studies regarding bone mineral changes and proximal migration of acetabular components this number of patients was considered adequate (Ornstein et al. 1999, Saari et al. 2014, Gerhardt et al. 2018, Zampelis et al. 2018). Multiple measurements were performed on each of them, so a linear model for repeated measurements was used to investigate factors influencing migration and bone mineral density changes. Within the linear model concept the fixed effects-only model with repeated statement was used. The functional form of the residuals was specified as compound symmetry for the covariance pattern. The degrees of freedom using the maximum likelihood method was obtained by Satterthwait approximation. P-values in Table 2 are a result of repeated measure ANOVAs. Statistical analyses were performed in SPSS Statistics 25 (IBM Corp, Armonk, NY, USA). A confidence interval (CI) of 95% was used.

**Table 2.** Postoperative measurements of bone mineral density in the ROIs. Values are mean (mg/cm³) with 95% confidence intervals

Region of interest	Postop., day 1	Time-point 6 weeks	2 years	p-value
Native bone				
cranial	137 (88–186)	145 (94–196)	132 (72 to 192)	0.2
caudal	79 (18–140)	93 (33–153)	35 (–38 to 108)	0.05
Bone graft	378 (355–401)	430 (412–448)	466 (390 to 542)	0.05

### Ethics, funding, and potential conflicts of interest

The study was approved by the Regional Ethics Committee, Uppsala, Sweden, Registration number 2015/228. The study received grants from the foundation Skobranschens utvecklingsfond and from the Center of Research and Development, Uppsala University, and County Council of Gävleborg. KB has a financial relationship with Mindways Software Inc. (Texas, USA). HO has a financial relationship with SECTRA AB (Linköping, Sweden). Other authors have no disclosures to make.

## Results

### CT-based migration

At 2 years, the translations were 1.5 mm (CI 0.4–2.6) in the proximal direction, –0.6 (CI –1.6 to 0.4) in the medial direction and 0.3 (CI 0.0–0.6) in the anterior direction (Table 1).

**Table 1.** Postoperative translations of the acetabular component. Values are mean (mm) with 95% confidence intervals

	Time-point	
Axis	6 weeks	2 years
X (med. +)	–0.16 (–0.46 to 0.14)	–0.56 (–1.56 to 0.44)
Y (prox. +)	0.31 (0.11 to 0.51)	1.52 (0.42 to 2.62)
Z (ant. +)	0.00 (–0.10 to 0.10)	0.27 (–0.03 to 0.57)

### Bone mineral density

Immediately postoperatively, the bone mineral densities in the 3 different regions of interest (ROIs) were: 378 mg/cm³ (CI 357–401) in the bone graft; 137 (CI 88–186) cranial to the graft; and 79 (CI 18–140) in the inferior ramus (baseline) (Table 2). Already at 6 weeks, density had increased 14% in the bone graft, continuing to 23% at 2 years (p = 0.05).

### Volume measurement

The mean bone grafted volume was 40 cm³ (CI 28–52) (Figure 2).

### Clinical results

Hip score according to Merle, d’Aubigne and Postel increased from 14 (CI 12–15) preoperatively to 18 (CI 18–18) postoperatively. There was 1 mechanical failure, which was excluded from the follow-up. The failure case had an AAOS type IV acetabular bone defect (pelvic dissociation), unknown at preoperative planning. The failure was aseptic with a proximal migration of 6 mm and a decrease in BMD in the bone graft, from 358 mg/cm³ postoperatively to 265 mg/cm³ at 2 years.

## Discussion

This study describes 2 new aspects: 1st, implant migration and bone mineral density can be measured simultaneously by low-dose CT scans. Instead of using 2 different labs, and 2 different examinations with 2 different machines, a low-dose standard CT scan can measure prosthetic migration and bone mineral density at the same time. 2nd, implant migration can be measured with high precision without tantalum markers in bone. In both primary and revision hip arthroplasty, a high proximal migration of acetabular components at 2 years predicts late loosening regardless of surgical technique (Klerken et al. 2015, Mohaddes et al. 2017b). Proximal migration and its predictive value is complex in revision surgery with bone grafting (Klerken et al. 2015, Mohaddes et al. 2017a). Comparisons are difficult because the severity of acetabular bone defects, graft preparation, impaction technique, and implants differs between studies. Our results of a proximal migration of 1.5 mm is comparable with previous reports (Ornstein et al. 1999, Mohaddes et al. 2017b), although 6 of our 17 cases had combined acetabular defects that required rim reinforcement. Zampelis et al. (2018) reported a proximal migration of only 0.22 mm—compared with 0.59 for their control group—at 2 years for cavitary defects when clodronate was added to the bone graft intraoperatively (Zampelis et al. 2018). In the same study, there was no statistically significant difference for the 2 groups in the bone mineral density (measured in the graft proximal to the cup) at 2 years.

Few studies exist on changes in bone mineral density during bone allograft incorporation (Gerhardt et al. 2018, Zampelis et al. 2018). DEXA measurements after hip revision with impaction bone grafting found an increase of 14% in the cranial graft bed after 2 years (Gerhardt et al. 2018). Our result of 23% at 2 years is rather close, and the difference may be because we measured with QCT instead of DEXA. The increase in actual trabecular bone mineral density in their study might also have been influenced by the fact that they included cortices in their measurement whereas we did not. At 6 weeks in our study, density increased in native bone cranial and caudal to the cup, although without statistical significance. In animal models, distant metaphyseal trauma affects mineralization and cellular expression in unrelated bones (Tatting et al. 2017). This effect plus increased load, mobilization, and metabolic activity may explain this unexpected finding in our study at 6 weeks. Postoperatively the graft bed was 3–5 times denser than the surrounding bone. Despite this, the density continued to increase in the graft during the follow-up period, both in our study and that of Gerhardt et al. (2018). This could be explained by further compression of dead bone graft rather than graft incorporation. However, of the total BMD increase in the bone graft, 59% occurred during the first 6 weeks but only 20% of the proximal migration occurred during the same period, indicating a biological response. Several studies of primary total hip arthroplasty show that cementless cup fixation decreases retro-acetabular bone stock more than cemented fixation (Wright et al. 2001, Digas et al. 2006). Differences in load distribution to the acetabulum may explain this, but fluid exchange and wear debris circulating through screw holes is also a possible cause (Fahlgren et al. 2010). Wright et al. (2001) have analyzed retro-acetabular bone mineral density changes with QCT 14 months after primary cementless cup fixation and found a decrease of 26% immediately cranial to the cup.

Goldvasser et al. (2012, 2014) have studied the accuracy of CT-based polyethylene wear measurements. They compared femoral head penetration on CT scans with measurements from a micrometer on the explanted liner after revision surgery. They found no statistically significant difference between the 2 methods, which emphasizes the accuracy of CT for wear measurements in vivo.

In our study only age affected the increase in bone mineral density and proximal migration (model linear regression). Bone graft volume, sex, and rim reinforcement did not.

Our study had some limitations. There were few patients, no inter- and intra-observer analysis of the measurements, and only 12 of the patients had double examinations performed. However, Schmidt et al. (2005) reported an inter- and intra-observer correlation coefficient over 0.99 in periacetabular osteo-densitometry, indicating a high reproducibility of QCT measurements around acetabular implants. Wright et al. (2001) also conducted intra-observer testing with a correlation coefficient of ≥ 0.89, although in a small group of 6 patients.

Our results also confirm previous results regarding BMD changes in bone graft (Gerhardt et al. 2018). The single failure case in our study had a pelvic discontinuity. He experienced a migration of 6 mm at 2 years and the second-largest bone grafted volume. We do not recommend the present method for patients with acetabular bone loss type IV (pelvic discontinuity).

Compared with plain radiographs a CT scan will enable:

1. prognosis prediction by migration analysis of the prosthetic implant;
2. wear measurements of implanted polyethylene cups or liners;
3. more accurate assessment of periprosthetic osteolysis and bone mineral density changes.

In conclusion, a postoperative, low-dose CT scan offers diagnostic potential. A radiation dose of 2.3 mSv is acceptable in this context.

The authors would like to thank Krister Ågren and Hans Högberg for statistical assistance.
